# N-butyl 2-Cyanoacrylate (NBCA) in Nephron Sparing Superselective Embolization of Iatrogenic Renovascular Injuries: A Single Centre Experience

**DOI:** 10.7759/cureus.33166

**Published:** 2022-12-31

**Authors:** Mohit Tayal, Khanak K Nandolia, Pankaj Sharma, Ankur Mittal, Udit Chauhan

**Affiliations:** 1 Radiodiagnosis, All India Institute of Medical Sciences, Rishikesh, Rishikesh, IND; 2 Urology, All India Institute of Medical Sciences, Rishikesh, Rishikesh, IND

**Keywords:** iatrogenic trauma, renal embolization, nbca, iatrogenic renal vascular injuries, selective trans-arterial embolization

## Abstract

Purpose

To evaluate the safety and efficacy of N-butyl-2-cyano-acrylate (NBCA) for endovascular management of iatrogenic renal-vascular injuries and effects on renal function.

Material & Methods

Fifteen patients with diagnosed or suspected iatrogenic renal vascular injuries, following percutaneous procedures formed the study group. All the patients were having retroperitoneal hemorrhage or hematuria, with hemodynamic instability at the time of presentation. Pseudoaneurysms, active extravasation of contrast, and the arteriovenous fistula were identified as the cause of bleeding on digital subtraction angiography. Patients underwent trans arterial super-selective embolization. Renal function was monitored using serum creatinine, estimated glomerular filtration rate (eGFR), and mean blood pressure of all the patients at immediate post-procedure and two months intervals.

Results

Technical and clinical success was achieved in all the cases using NBCA alone. Patients improved hemodynamically. None of the patients required repeat embolization. No derangement in renal function was observed immediately after the procedure and at interval follow-up.

Conclusion

NBCA can be used as a safe embolizing agent to provide a quick and effective cure for iatrogenic renovascular injuries. Renal parenchymal loss can be minimized by super selective technique.

## Introduction

Renal vascular injury can be traumatic or iatrogenic, following surgery or a percutaneous renal intervention [1-4]. American Association for the Surgery of Trauma (AAST) classifies renal injuries as per the renal injury scale. Vascular injuries are added in the 2018 version of the classification. Vascular injury or active bleeding limited by the perirenal fascia is a grade III injury. Grade IV injury spectrum includes segmental renal vessel injury, segmental infarction, and active bleeding beyond perirenal fascia. Grade V injury is hilar vascular injury, which may lead to renal devascularization. Vascular injuries can be self-limiting or life-threatening hemorrhages leading to persistent hematuria and hemodynamic instability, warranting emergent management [5]. 

The minimally invasive nature makes endovascular embolization an appropriate alternative to open surgeries. Indications of endovascular management include massive bleeding, expanding retroperitoneal and perinephric hematoma, non-resolving gross hematuria, and continuous requirement of hemodynamic support [6]. Super-selective catheterization with microcatheters is important for renal parenchymal preservation [1, 7]. Conventionally, coils were the preferred embolic agents to treat reno-vascular injuries. Drawbacks of coiling are unpredictable embolization outcome, dependence on the native coagulation pathways, cost of materials, and limited availability of appropriately sized coils [8, 9]. N-butyl cyanoacrylate (NBCA) commonly known as glue, a liquid embolic agent, emerged as a promising agent providing immediate, safe, and effective devascularization [10-13]. This study was intended to assess the safety and efficacy of using NBCA as a primary embolic agent to treat iatrogenic renal vascular injuries.

## Materials and methods

A retrospective analysis of medical records of patients who underwent renal artery embolization following iatrogenic injuries, between March 2020 and March 2022, was performed. Approval from the Institutional ethical committee was obtained. All the procedures were performed after written informed consent. Cases of spontaneous and tumoral hemorrhage were excluded. None of the patients had absolute or relative contraindications for embolization or angiography.

Detailed evaluation of pre-procedure clinical features and existing co-morbidities like chronic renal disease was done. Computed tomography (CT) images were reviewed in detail to ascertain underlying etiologies. Coagulation parameters like platelet counts and international normalized ratio, and renal function parameters like serum creatinine and estimated glomerular filtration rate (eGFR) were evaluated.

Procedures were performed using a digital biplane angiographic unit (Allura, Philips, Netherlands). All procedures were done under anesthesia support, consisting of mild sedation or general anesthesia when required. Vital parameters were monitored in all the cases. Under local anesthesia, the right common femoral artery was accessed using the Seldinger technique with a 5 Fr arterial short sheath (Boston Scientific, Boston, MA, USA) under ultrasound guidance. Cobra-2 (Cook Medical, Bloomington, IN, USA) angiographic catheter was used to cannulate the renal arteries. Sim-1 (Simmons) and Shepherd hook (Boston Scientific, Boston, MA, USA) catheters were reserved for difficult and tortuous anatomy. A renal artery angiogram was performed using a non-ionic iodinated contrast agent (iobitridol 350 mg/ml Xenetix, Guerbet, France). Baseline angiograms were performed to identify the vascular anatomy and to characterize and localize the vascular injury. After identifying the vascular injury, super selective catheterization with a co-axial system consisting of a 2.7 Fr microcatheter (Progreat, Terumo, Somerset, NJ, USA) over the microwire was performed up to the interlobular arteries. Efforts to preserve maximum renal parenchyma were made by advancing the tip of the catheter, as distally as possible, under fluoroscopic guidance. Adequate dose and injection rate of embolization agent were cautiously determined to avoid non-target embolization due to reflux. The dilution ratio of NBCA (Histoacryl, Braun Surgical, SA, Rubi, Spain) with ethiodized poppy-seed oil (Lipiodol; Andre Guerbet, Aulnay-Sous-Bois, France) was between 1:2 and 1:4. Ratio was determined by the distance of target lesion from the tip of microcatheter, the flow rate in the vessel and, absence or presence of arterio-venous shunting. Microcatheter coaxial assembly was manually flushed with Dextrose 5% in water (D5W) prior to NBCA mixture injection. NBCA - lipiodol mixture was carefully injected under image guidance to observe complete embolization and to avoid reflux. The microcatheter was subsequently removed in a single quick snap. A post-embolization arteriogram was performed to confirm the embolization endpoint. The technical and clinical outcomes of the embolization were registered. Technical success was defined as cessation of ongoing bleeding from renal vessels and stable renal function post-embolization. Preservation of renal parenchyma was measured by comparing parenchymal phases of pre-embolization and immediate post-embolization angiograms. Hemostasis was achieved at the puncture site using manual compression.

Clinical success was ascertained by stable hemoglobin levels, resolving hematuria/hematoma, reduced need for blood transfusion, and hemodynamic stability.

Statistical analysis

The data were entered in Microsoft Excel and analyzed using IBM SPSS Statistics for Windows, version 21.0 (IBM Corp., Armonk, N.Y., USA). Continuous variables were expressed as mean ± standard deviation (SD). Categorical data were expressed in frequency and proportion. One-way ANOVA with Post hoc Bonferroni correction was applied for the relationship between laboratory parameters, pre, and post-procedure. A “p” value less than 0.05 was considered statistically significant.

## Results

Complete cessation of bleeding with control of the bleeding source was achieved in all cases. Table 1 details the demographic and clinical data of all the cases. Fifteen patients with a mean age of 30.87 ± 7.3 years (minimum 19, maximum 42 years), distributed in male to female ratio of 3:2 formed the study group.

**Table 1:** Demographic data and clinical characteristics of patients included in the study. M: Male, F: Female, PsA: Psuedoaneurysm, AL: Active Leak, L: Lower pole, U: Upper Pole, HDN: Hydronephrosis, DM: Diabetes Mellitus, ADPKD: Autosomal Dominant Polycystic Kidney Disease, PCN: percutaneous nephrostomy, PCNL: percutaneous nephrolithotomy.

Case No	Age	Gender	Source of bleed	Site	Cause of iatrogenic injury	Comorbidity
1	23	M	PsA	L	PCNL	None
2	29	M	AL	L	PCNL	None
3	21	M	AL	L	PCNL	None
4	19	M	PsA + AL	L	PCNL	None
5	32	M	AL	L	PCN	None
6	35	F	PsA	U	PCNL	HDN
7	37	F	PsA	L	Biopsy	ADPKD
8	31	M	PsA	L	PCN	None
9	42	F	AL from capsular artery	U	Biopsy	DM
10	20	M	PsA	U	PCNL	None
11	39	M	AVF	L	Biopsy	None
12	40	F	AL	L	Biopsy	DM
13	37	F	PsA	L	PCNL	None
14	28	M	PsA	U	PCNL	None
15	30	M	PsA	U	PCNL	None

Percutaneous nephrolithotomy (PCNL) was the most common (n=9, 60%) cause of iatrogenic renal vascular injury in our cohort, followed by percutaneous biopsy (n=4, 26.7%) and percutaneous nephrostomy (n=2, 13.3%).

All the bleeding sources were from the subsegmental renal vessels, making them AAST grade III injuries. Proximal, hilar, or segmental renal vessel injuries were absent in all the cases. None of the cases in our study was AAST grade V renal injury. No renal hilar injuries or total devascularization was observed.

According to the source of bleeding, we will discuss our cases in three subgroups - pseudoaneurysms, active direct arterial extravasation, and arterio-venous fistula.

**Figure 1:**
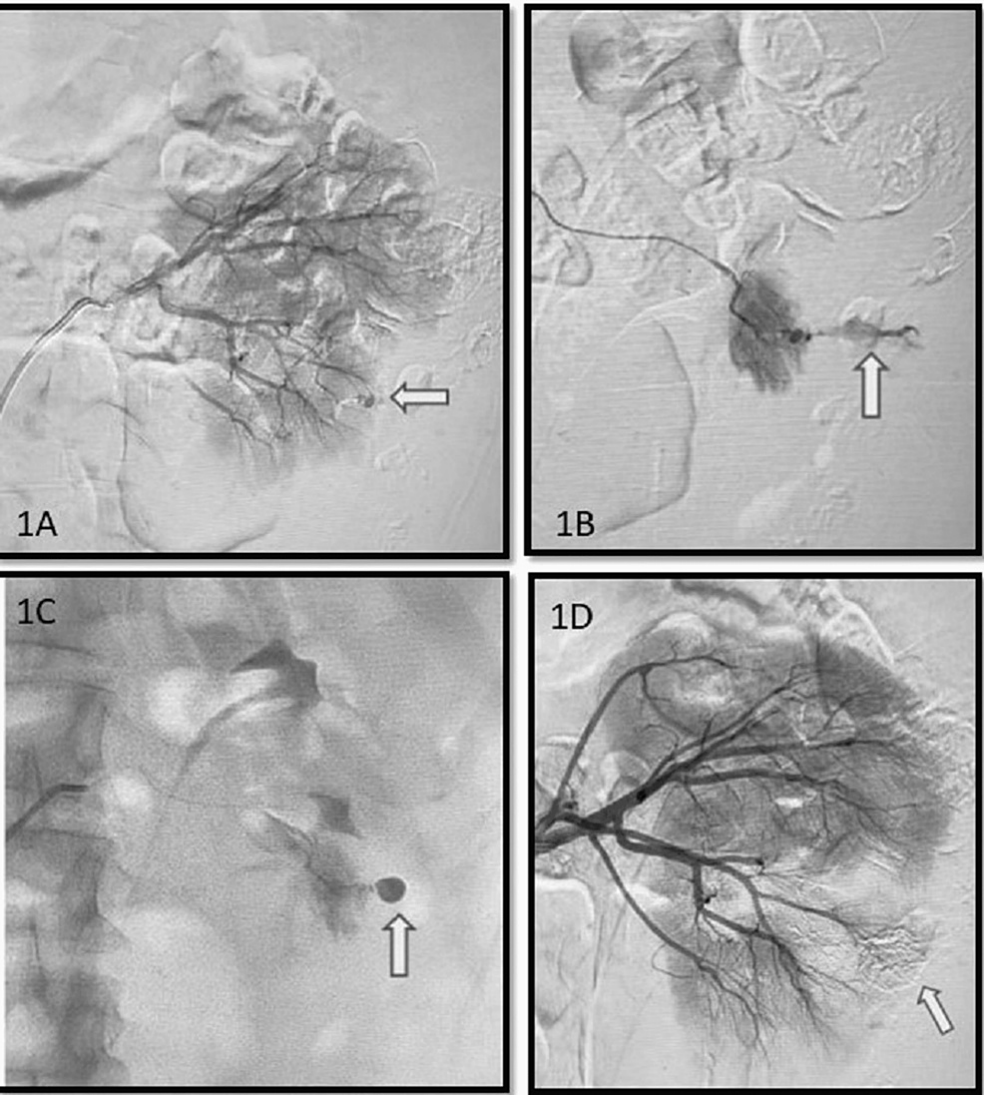
A case of post PCNL left renal injury in a 19-year-old male. Proximal angiogram (A) shows a small pseudoaneurysm sac (arrow) at the lower pole subsegmental artery. Superselective microcatheter angiogram shows active extravasation (B) of the contrast medium (arrow). After super-selective micro-catheterization, sac was completely embolized with NBCA (C). Post embolization angiographic parenchymal phase shows NBCA cast, <25% devascularization and complete cure of extravasation (D).

Solitary pseudoaneurysm (Figure 1) was present in nine cases (60%), making it the most frequently encountered abnormality on DSA. The most common cause of pseudoaneurysm formation was percutaneous nephrolithotomy (PCNL) in our study with seven cases. Two pseudoaneurysm cases were post percutaneous nephrostomy (PCN) placement and one pseudoaneurysm was post-renal biopsy in a case of autosomal dominant polycystic kidney disease. Pseudoaneurysms were seen in the upper pole arteries in five cases and in the lower pole arteries in four cases. These cases showed small focal abnormal arterial dilatation with associated contrast extravasation. Pseudoaneurysms required distal super-selective cannulation with precise treatment. Proximal embolization was avoided. Distal collateral flow can recanalize the aneurysm after proximal embolization. Hence, direct embolization of the pseudoaneurysm was done in all cases.

**Figure 2:**
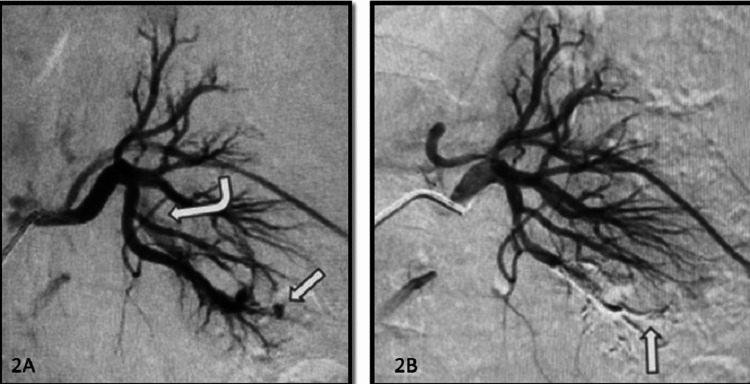
A case of post biopsy left renal injury in a 39-year-old male. Proximal angiogram in arterial phase (A) shows contrast blush (straight arrow) from a lower pole subsegmental artery. Lower pole segmental vein shows early opacification in arterial phase (curved arrow) due to fistula formation. Post NBCA embolization angiogram in arterial phase (B) shows cast formation (straight arrow) with <10% devascularization. Contrast blush and arterio venous fistula are cured.

One post-biopsy patient had an arteriovenous fistula at the lower pole (Figure 2). The arteriovenous fistula was characterized by the presence of an early draining vein in the arterial phase of the injection. The fistula site as well as the distal part of the arterial limb of the fistula were embolized.

**Figure 3:**
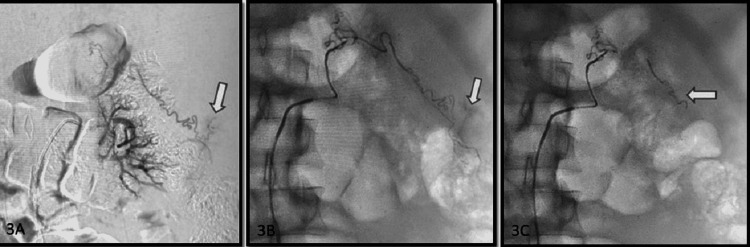
A case of post biopsy left renal injury in a 42-year-old female. Angiographic images in late arterial phase with proximal (A) and distal super selective microcatheter (B) angiograms show active contrast extravasation (arrow) from the upper pole capsular branch. Post NBCA embolization, microcatheter angiogram (C) shows no extravasation at the site of injury (arrow).

Active direct arterial contrast extravasation without associated pseudoaneurysm on the angiographic arterial phase was present in six (40%) cases. Among the cases, three cases were post-PCNL (Figure 3). Two cases were post renal biopsy and one case was post PCN placement. Five cases showed active leaks from lower pole arteries, while one case showed upper pole artery injury. An active contrast leak was seen from the subcapsular artery in one case. Active contrast leak cases were characterized by extravasation of contrast from the site of instrumentation or intervention. Blotchy contrast pooling was seen at the termination of a culprit artery.

Technical and clinical success was achieved in all the cases. Repeat embolization or surgery was not required in any of the cases within two months of follow-up. The renal parenchymal loss was limited to less than 25% in all the cases.

The follow-up of all patients was done two months after embolization. Serum creatinine (SCr), eGFR, and mean blood pressure were measured as markers of renal function. Their values at two months follow-up were compared with the pre-procedure and post-procedure first-day values. 

Table 2 details these values at different time points. Differences between the values of SCr, eGFR and mean blood pressure obtained at different time periods were not statistically significant, with p values 0.71, 0.99, and 0.97 respectively on the application of one-way ANOVA. There was no association seen between the groups on the post hoc test, Bonferroni correction.

**Table 2:** Pre and post-embolization comparison of direct and indirect renal function parameters. eGFR - estimated glomerular filtration rate, mmHg - millimeters of Mercury

Parameter	Pre-procedure	Immediate post procedure	2 month Follow up	One-way ANOVA p ‘value’
Mean serum creatinine (mg/dl)	1.01±0.28	1.01±0.18	0.99±0.19	0.71
Mean eGFR (ml/min/1.73m^2^)	99.4±7.19	99.53±6.86	99.2±7.12	0.99
Mean blood pressure (mm Hg)	90.67±7.99	90.93±8.13	90.27±8.64	0.97

## Discussion

Iatrogenic renal vascular injuries during percutaneous procedures are common. Percutaneous nephrolithotomy was the commonest cause in our cohort, followed by percutaneous biopsy and nephrostomy. Patients may present with immediate or persistent delayed hematuria, deteriorating vitals, or renal function. Patients may have a duration of symptoms between a few hours and few days. In our study, all the patients were referred for angiography or embolization within a few hours to a few days of the intervention during their post-procedure hospital stay itself. The decision between continuing conservative management and opting for surgical or endovascular management largely follows the general condition of the patient. Blood transfusion is required in 1 to 11% of such cases Hemodynamically stable patients are intervened upon the persistence of hematuria >72 hours and/or renal function decline. Hemodynamic instability requires immediate intervention. The spectrum of angiographic findings in iatrogenic renal injuries includes pseudoaneurysms, active extravasation, and arteriovenous or arterio-calyceal fistula [2, 14-17].

Iatrogenic renal vascular injuries usually manifest as a solitary bleeding source. Multiple bleeding sources are generally found with systemic pathologies resulting in spontaneous hemorrhage. Trans arterial embolization (TAE) is an effective alternative to surgery in the management of renovascular injuries after the failure of conservative measures. TAE has shorter post-procedure hospital stays; and lower morbidity as compared to open surgeries. TAE can reduce the need for blood transfusions. The availability of microcatheters and coaxial systems has facilitated ‘nephron sparing’ super-selective embolization [15]. Early and accurate diagnosis can defer nephrectomy [11, 18-20]. Trans-arterial embolization is not devoid of complications. Post-embolization syndrome, characterized by abdominal pain, mild fever, and nausea, is the most commonly encountered complication. However, it is usually self-limiting and can be managed conservatively. Five patients (33.3%) patients in our study developed the post-embolization syndrome.

The choice of an embolizing agent is crucial for technical success and depends upon the preference and expertise of the endovascular specialist; and various patient factors like vascular anatomy, hemodynamics, pathology, and desired endpoint.

Endovascular coils are placed proximally to reduce or obliterate blood flow. Particulate agents like polyvinyl alcohol particles, liquid agents like NBCA, and sclerosants are used for end-artery embolization and disruption of the vascular bed [14].

Polyvinyl alcohol particles and coils obliterate the vessel lumen by inducing vasculitis and thrombosis. Multiple coils deployment can increase the cost and length of the procedure. The unavailability of appropriately sized coils at the time of embolization can delay the procedure. Particulate agents can cause systemic and pulmonary embolization in cases of post-traumatic AV fistula [16].

NBCA, due to its liquid nature, percolates distally into the target vessels. NBCA polymerizes and rapidly precipitates upon contact with an ionic fluid medium- such as blood. Hence, NBCA can be effectively used in patients with systemic coagulopathy, as its functioning is independent of the coagulation cascade [15, 16]. The composition of the NBCA - lipiodol mixture determines the rapidity of polymerization and the distance traveled by the mixture in the target vessel. The higher concentration is used in high-flow lesions and for proximal rapid precipitation. Lower concentration is preferred to allow gradual distal percolation and complete distal embolization, which prevents subsequent recanalization [1, 16].

Despite the good clinical outcomes, the use of NBCA is technically demanding. Too high a concentration of NBCA can cause proximal early polymerization and subsequent catheter tip trapping or adherence to the vessel wall. NBCA mixture residues in the catheter assembly can cause non-target or shower embolization due to inadvertent flushing while obtaining post-embolization check angiograms and during retrieval of the coaxial system [21-23]. No similar complications were encountered in our study as we followed due precautions.

The use of NBCA for the management of iatrogenic renovascular injuries has been scarcely reported in the literature [21-24]. Our experience of complete technical and clinical success with NBCA embolization was comparable with the results obtained by Mavilli et al. (4/4), Joon et al. (12/14), and Cantasdemir et al. (5/5) in their respective studies [10, 11, 16].

Embolization and endovascular interventions have their own drawbacks - such as femoral puncture site complications, vascular dissections, and post-embolization syndrome. However, their incidence remains fairly low. Organ-specific complications include non-selective or proximal renal artery embolization, leading to renal function loss due to large-volume parenchymal infarction and subsequent renal scarring [16, 20]. However, in our study, super selective catheterization with microcatheters up to the level of segmental renal vessels avoided inadvertent proximal embolization and preserved normal renal parenchyma. The use of microcatheters causes approximately 10% loss of renal parenchyma, but not more than 25%, provided the pathology is limited to a single pole of the kidney. All of our patients had stable and normal serum creatinine and eGFR values immediately following the embolization and in two months follow-up. A case of post-PCNL bleed with hydronephrosis showed a decline in serum creatinine from 1.7 mg/dl (pre-procedure) to 0.9mg/dl at two-month follow-up post-TAE and double-J stenting.

Incomplete proximal embolization and subsequent partial recanalization result in a patent, but stenotic native renal arteries. Further recruitment of lumbar collaterals can lead to renal vascular hypertension and renal functional decline [2, 7, 25 - 27]. None of our cases developed post-embolization hypertension at the two month follow-up. This can be attributed to super selective distal vascular access for embolization. None of the cases in our study had a single kidney or a transplanted kidney.

Selective renal embolization may not lead to favorable outcomes or cures in all cases. As per the study by Baboudjian et al., factors predicting embolization failure are - gross hematuria, hemodynamic instability, grade V injury, and extravasation of urine. However, embolization may still be preferred in these cases. Selective embolization was found to be a good alternative to surgery or nephrectomy according to this large multicentre trial [28].

Our study has limitations and bias owing to its retrospective nature that introduced inevitable selection bias. Another limitation is the small patient cohort involved in this study, and thus the results need to be validated in a larger setting. Serum creatinine, eGFR and mean blood pressure were used as surrogate markers of renal function, which may not be accurate enough. We determined renal parenchymal loss based on the parenchymal phase of post-embolization angiograms.

Our work will be of substantial addition to the previously reported articles as it is the first of its kind in the setting of iatrogenic renal injuries. With the growing number of percutaneous procedures, iatrogenic injuries may come as a common clinical encounter.

## Conclusions

Iatrogenic renal vascular injuries are common in multi-disciplinary hospitals and often they require resuscitation and acute management. A minimally invasive endovascular treatment option remains an attractive option with less morbidity as compared to open surgeries. N-butyl cyano-acrylate (NBCA) embolization can control iatrogenic renal hemorrhages. Super selective embolization with NBCA limited the extent of renal infarction and preserves the maximum possible renal function in our study.
